# Conditional survival nomogram predicting real-time prognosis of locally advanced breast cancer: Analysis of population-based cohort with external validation

**DOI:** 10.3389/fpubh.2022.953992

**Published:** 2022-10-31

**Authors:** Xiangdi Meng, Furong Hao, Zhuojun Ju, Xiaolong Chang, Yinghua Guo

**Affiliations:** ^1^Department of Radiation Oncology, Weifang People's Hospital, Weifang, China; ^2^Department of General Medicine, Weihai Central Hospital, Weihai, China

**Keywords:** locally advanced breast cancer, conditional survival, predictors, nomogram, prognosis

## Abstract

**Background:**

Locally advanced breast cancer (LABC) is generally considered to have a relatively poor prognosis. However, with years of follow-up, what is its real-time survival and how to dynamically estimate an individualized prognosis? This study aimed to determine the conditional survival (CS) of LABC and develop a CS-nomogram to estimate overall survival (OS) in real-time.

**Methods:**

LABC patients were recruited from the Surveillance, Epidemiology, and End Results (SEER) database (training and validation groups, *n* = 32,493) and our institution (testing group, *n* = 119). The Kaplan–Meier method estimated OS and calculated the CS at year (x+y) after giving x years of survival according to the formula CS(y|x) = OS(y+x)/OS(x). y represented the number of years of continued survival under the condition that the patient was determined to have survived for x years. Cox regression, best subset regression, and the least absolute shrinkage and selection operator (LASSO) regression were used to screen predictors, respectively, to determine the best model to develop the CS-nomogram and its network version. Risk stratification was constructed based on this model.

**Results:**

CS analysis revealed a dynamic improvement in survival occurred with increasing follow-up time (7 year survival was adjusted from 63.0% at the time of initial diagnosis to 66.4, 72.0, 77.7, 83.5, 89.0, and 94.7% year by year [after surviving for 1–6 years, respectively]). In addition, this improvement was non-linear, with a relatively slow increase in the second year after diagnosis. The predictors identified were age, T and N status, grade, estrogen receptor (ER), progesterone receptor (PR), human epidermal growth factor receptor 2 (HER 2), surgery, radiotherapy and chemotherapy. A CS-nomogram developed by these predictors and the CS formula was used to predict OS in real-time. The model's concordance indexes (C-indexes) in the training, validation and testing groups were 0.761, 0.768 and 0.810, which were well-calibrated according to the reality. In addition, the web version was easy to use and risk stratification facilitated the identification of high-risk patients.

**Conclusions:**

The real-time prognosis of LABC improves dynamically and non-linearly over time, and the novel CS-nomogram can provide real-time and personalized prognostic information with satisfactory clinical utility.

## Introduction

Locally advanced breast cancer (LABC) is a relatively advanced stage of breast cancer, but benefitting from advances in diagnosis and treatment strategies, the overall survival (OS) has improved in recent years (1–3). Therefore, more and more long-term survivors hoped to know more accurate prognostic information during follow-up, but LABC still lacked effective prognostic assessment tools. Indeed, the risk of death in cancer patients should be dynamic and gradually improve with follow-up time (4–8). This favorable survival feature has long been widely used in many cancers but not in LABC.

In cancer follow-up, the strong impact of long-term survival on subsequent survival is known as conditional survival (CS) (9, 10), defined as the probability of surviving further y years, given that a patient has already survived x years after the diagnosis of a disease (11). It is possible to evaluate the improvement of the survival rate after surviving for several years. This characteristic may give patients and researchers more critical practical clinical prognosis information. Studies on malignant tumors of the head and neck (12, 13), lung (14, 15), esophagus (7, 16), stomach (5), colorectal (17, 18), and other areas (4, 6, 19) demonstrated that the OS of cancer changes dynamically over time. The CS may decline rapidly in the first 1–2 years and then stabilize (17). So far, it has yet to be investigated whether this feature existed in LABC.

Precise dynamic risk assessment can correctly identify LABC patients at risk of death, and high-risk patients may be candidates for more frequent follow-up or clinical trials. Although CS could calculate a more accurate prognosis than traditional survival analysis, individual factors were not considered. At the same time, general nomograms integrated individual predictors but did not consider survived time, which might also hinder accurate assessments. Suppose we combined the nomogram with CS to develop a novel dynamic prediction model. In that case, it would be possible to simultaneously have the advantages of dynamic, personalization, and accuracy.

This study aimed to estimate the CS of LABC and develop a dynamic CS-nomogram to accurately assess OS in real-time and identify high-risk patients, providing valuable information for survival assessment and follow-up.

## Materials and methods

### Data sources, patient selection and variables

Data in this study were obtained from Weifang People's Hospital and 18 regional population-based registries in the Surveillance, Epidemiology, and End Results (SEER) database released in 2020. Before using the SEER database, we signed the SEER Research Data Use Agreement and obtained access (username: 15029-Nov2020). In this retrospective study, patient data were anonymized; thus, our study was exempted from the Institutional Review Board review.

At M.D. Anderson Cancer Centre, LABC was defined as a primary tumor >5 cm or any diameter with chest wall or skin involvement and/or lymph node status was N2-3 (20). Therefore, we selected breast cancer patients diagnosed with T3N0M0 and stage III disease from the SEER database (2010–2017) and our medical center and removed available variables [age, primary tumor location, American Joint Committee on Cancer (AJCC) tumor-node-metastasis (TNM) stage (8th edition), immunohistochemical information, histological grade, surgery and chemoradiotherapy information, survival status and time] were unknown. Notably, the definitions of T3N0M0 and stage III were consistent in AJCC 7th (used from 2010 to 2015) and AJCC 8th (used from 2016 to 2017), and therefore no adjustment of staging was required. According to the definition of elderly and the age at high-risk of breast cancer, the age was divided into groups ≤35, 36–70, and >70 years (21). In addition, data that were not histopathologically confirmed, non-first primary cancer, follow-up time of 0 months, and data on beam radiation not used in radiotherapy patients were also excluded from our consideration. Moreover, the clinical endpoint of the study was OS, defined as the time between patient's diagnosis and death from all causes.

### Statistical analysis

Based on the experience of previous studies and the larger sample size of this study (22–25), the data screened from the SEER database were divided into training and validation groups in a ratio of 2:1, and the data from our institution was used as the testing group. Categorical variables were counted and reported as percentages, and chi-square or fisher's exact tests were performed to compare differences.

The OS was calculated using the Kaplan–Meier method. The CS was calculated by the formula CS(y|x) = OS(y+x)/OS(x) (10, 11), where CS(y|x) indicated the probability of surviving further y years, if the patient has already survived x years after the LABC diagnosis. OS(y+x) and OS(x) indicated the (y+x) and x year OS calculated by the Kaplan-Meier method, respectively. For example, CS(2|3) =OS(2+3)/OS(3) was the probability of 5 years CS that a patient who survived for a further 2 years after surviving for 3 years after initial diagnosis.

This study used three methods to screen predictors, including univariate Cox regression (*p* < 0.05 as screening criteria), the least absolute shrinkage and selection operator (LASSO) regression plus 5-fold cross-validation (one standard error of the minimum mean square error is used as a screening criterion), and best subset regression (BSR) (adjusted R-squared maximum as screening criteria). The subset of variables screened by these three methods were then subjected to the multivariate Cox regression with stepwise backward regression to further determine the final model, which was determined by the Akaike information criterion (AIC) minimum and R-squared maximum, which ensured the goodness of fit of the model.

A nomogram was built based on the optimal model described above. All variables in the nomogram were quantified as points. When a patient entered the prognostic features, the total risk points would be calculated, corresponding to an individualized survival rate. Additionally, under the CS formula [CS(y|x) = OS(y+x)/OS(x)], the real-time survival at year 7 after surviving several years after diagnosis (e.g., 1–6 years) could be found under this score. In addition, the web version of this nomogram made it easier to use. The maximum standardized log-rank statistic was used to find the optimal cut-off point for classification into the high-risk and low-risk groups based on the total score for all patients calculated by the nomogram. The Kaplan–Meier method was used to assess the difference in OS between the two groups of patients.

Model performance was evaluated and validated in training, validation, and testing groups. The concordance index (C-index) and time-dependent receiver operating characteristic (ROC) were used to assess discrimination. Calibration plots with 1,000 bootstrap samples were used to observe the model's accuracy. The clinical usefulness of the nomogram was assessed by decision curve analysis (DCA) which assessed the net benefit of nomogram-guided medical interventions. The statistical analysis of this study was performed in March 2022 using R (version 4.1.0). *P*-values < 0.05 were considered statistically significant in the two-tailed test.

## Results

### Clinicopathological characteristics

**Figure 1:**
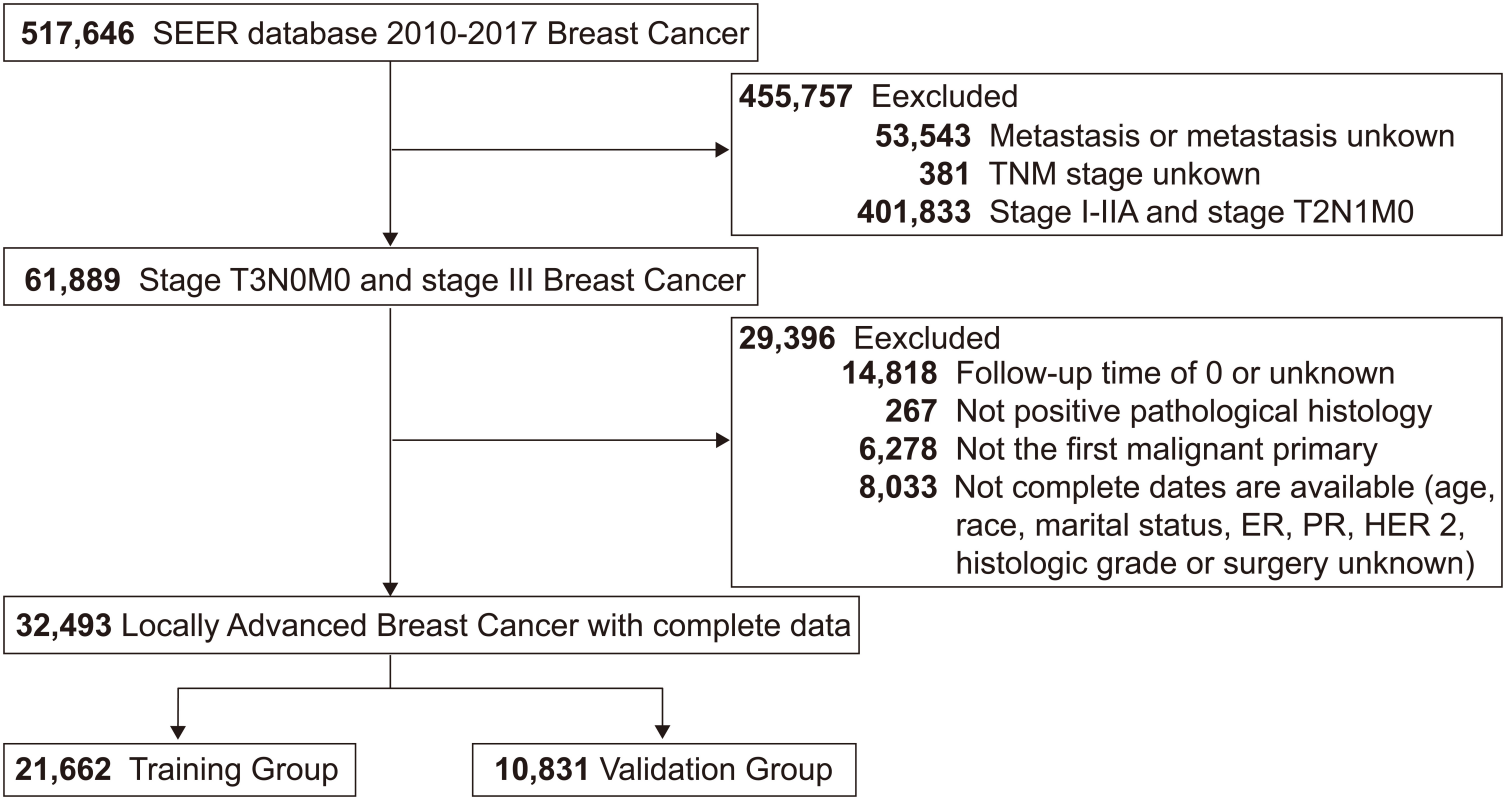
Study flowchart of patients with locally advanced breast cancer (LABC) from the Surveillance, Epidemiology, and End Results (SEER) database. Bold values indicate the number of patients.

This study screened 517,646 patients diagnosed with breast cancer from 2010 to 2017 from the SEER database, and 32,493 LABC patients who met the inclusion-exclusion criteria were finally divided 2:1 into a training group (*N* = 21,662) and a validation group (*N* = 10,831). The patient screening process was shown in Figure 1. Meanwhile, the number of LABC patients in the testing group was 119. For the entire cohort, the mean age [standard deviation (SD)] was 58 (14.5) years, and the mean follow-up time (SD) was 48.3 (28.4) months. All variables in the training and validation groups were not significantly different, while the primary site, TNM stage, and radiotherapy in the testing group were different from those in the training group, possibly due to differences in geographical and treatment strategy. See Table 1 for details.

**Table 1:** Patient clinicopathologic characteristics in locally advanced breast cancer.

**Characteristics**	**Training group**	**Validation group**	**Test group**	***P*** **-value^a^**	***P*** **-value^b^**
	**n = 21,662 (%)**	**n = 10,831 (%)**	**n = 119 (%)**		
Age (years)				0.955	0.229
≤35	1,012 (4.67)	498 (4.60)	3 (2.52)		
36–70	16,160 (74.60)	8,089 (74.68)	85 (71.43)		
>71	4,490 (20.73)	2,244 (20.72)	31 (26.05)		
Primary site				0.241	<0.001
Upper outer	7,046 (32.53)	3,615 (33.38)	67 (56.30)		
Lower outer	1,395 (6.44)	648 (5.98)	6 (5.04)		
Upper inner	1,683 (7.77)	834 (7.70)	15 (12.61)		
Lower inner	824 (3.80)	372 (3.43)	4 (3.36)		
Center	1,597 (7.37)	794 (7.33)	5 (4.20)		
Other	9,117 (42.09)	4,568 (42.18)	22 (18.49)		
Primary laterality				0.984	0.164
Left	10,974 (50.66)	5,485 (50.64)	50 (42.02)		
Right	10,677 (49.29)	5,341 (49.31)	69 (58.98)		
Other	11 (0.05)	5 (0.05)	0 (0.00)		
T status (AJCC-8th)				0.732	<0.001
T0	29 (0.13)	13 (0.12)	1 (0.84)		
T1	2,312 (10.67)	1,120 (10.34)	10 (8.40)		
T2	5,870 (27.10)	2,907 (26.84)	15 (12.61)		
T3	9,117 (42.09)	4,638 (42.82)	80 (67.23)		
T4	4,334 (20.01)	2,153 (19.88)	13 (10.92)		
N status (AJCC-8th)				0.264	0.009
N0	3,950 (18.23)	1,941 (17.92)	9 (7.56)		
N1	5,315 (24.54)	2,761 (25.49)	30 (25.21)		
N2	7,948 (36.69)	3,900 (36.01)	57 (47.90)		
N3	4,449 (20.54)	2,229 (20.58)	23 (19.33)		
TNM stage (AJCC-8^th^)				0.917	<0.001
T3N0M0	2,973 (13.72)	1,461 (13.49)	8 (6.72)		
IIIA	10,692 (49.36)	5,380 (49.67)	80 (67.23)		
IIIB	3,548 (16.38)	1,761 (16.26)	8 (6.72)		
IIIC	4,449 (20.54)	2,229 (20.58)	23 (19.33)		
Histologic grade				0.835	0.704
I-II	10,998 (50.77)	5,513 (50.90)	63 (52.94)		
III-IV	10,664 (49.23)	5,318 (49.10)	56 (47.06)		
ER				0.917	0.988
Negative	5,357 (24.73)	2,672 (24.67)	30 (25.21)		
Positive	16,305 (75.27)	8,159 (75.33)	89 (74.79)		
PR				0.544	0.959
Negative	7,968 (36.78)	3,946 (36.43)	43 (36.13)		
Positive	13,694 (63.22)	6,885 (63.57)	76 (63.87)		
HER 2				0.439	1.000
Negative	16,951 (78.25)	8,434 (77.87)	93 (78.15)		
Positive	4,711 (21.75)	2,397 (22.13)	26 (21.85)		
Molecular subtypes				0.58	0.828
Luminal A	13,553 (62.57)	6,793 (62.72)	78 (65.55)		
Luminal B	3,054 (14.10)	1,544 (14.26)	17 (14.29)		
HER2 enriched	1,657 (7.65)	853 (7.88)	9 (7.56)		
Triple negative	3,398 (15.69)	1,641 (15.15)	15 (12.61)		
Surgery				0.751	0.393
No	1,745 (8.06)	889 (8.21)	10 (8.40)		
BCS	4,566 (21.08)	2,249 (20.76)	19 (16.97)		
Mastectomy	15,351 (70.87)	7,693 (71.03)	90 (75.63)		
Radiotherapy				0.083	<0.001
No/unknown	7,599 (35.08)	3,906 (36.06)	22 (18.49)		
Yes	14,063 (64.92)	6,925 (63.94)	97 (81.51)		
Chemotherapy				0.789	0.614
No/unknown	4,880 (22.53)	2,455 (22.67)	24 (20.17)		
Yes	14,782 (77.47)	8,376 (77.33)	95 (79.83)		

a Training group vs. validation group;

b Training group vs. test group.

AJCC-8th, American Joint Committee on Cancer (8th Edition); TNM stage, tumor-node-metastasis stage; ER, estrogen receptor; PR, progesterone receptor; HER 2, human epidermal growth factor receptor 2; BCS, breast conservation surgery.

### Conditional survival analysis of LABC

**Figure 2:**
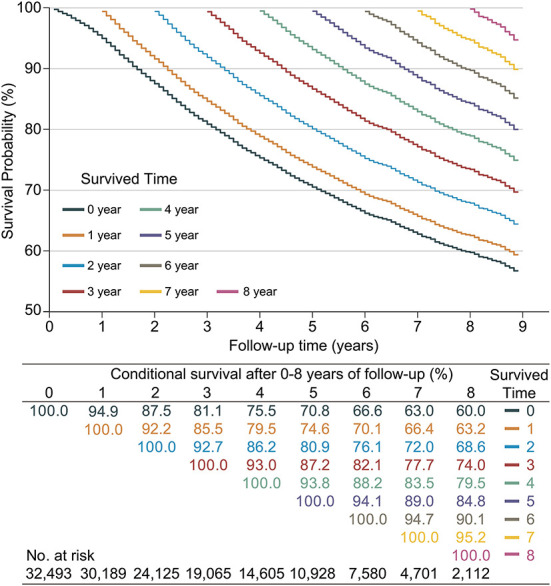
Kaplan–Meier method for estimating conditional survival (CS) of locally advanced breast cancer (LABC) at given years of survival. Each curve represented to the survival rate after a given number of years (e.g., the orange curve indicated the survival after given 1 year of follow-up [CS(y|1)]), which corresponded to the orange row in the table, i.e., after given 1 year of follow-up, survival in the 2nd year was 92.2% [CS(1|1)] and 66.4% [CS(6|1)] in the 6th year; Additionally, each column in the table represented the patient's survival after given different follow-up times, e.g., the survival in year 8 was adjusted from an initial 60.0% [CS(8|0)], gradually adjusted to 95.2% [CS(1|7)].

Since CS analysis required a large amount of data, only the SEER database was used for this analysis. A total of 8,702 LABC patients (26.78%) died in the SEER database. The 3, 5, and 7 year OS rates for LABC were 81.1% (95% confidence interval [CI]: 80.7–81.6%), 70.8% (95% CI: 70.2–71.4%), and 63.0% (95% CI: 62.3–63.7%), respectively. The CS Kaplan-Meier curve showed that the real-time survival rate of LABC dynamically increased over time (Figure 2). The 7 year survival rate of patients was adjusted from 63% at the initial diagnosis to 66.4, 72.0, 77.7, 83.5, 89.0, and 94.7% year by year (after surviving for 1–6 years, respectively).

At the same time, we found that if the patients survived for 2 years, their next-year survival rate [CS(1|x)] gradually increased for each additional year they lived. Specifically, patients had the lowest 1 year CS in the second year of follow-up, with CS (1|1) = 92.2%. This value gradually increased over the subsequent follow-up time, namely CS(1|2) = 92.7%, CS(1|3) = 93.0%, CS(1|4) = 93.8%, CS(1|5) = 94.1%, CS(1|6) = 94.7%, and CS(1|7) = 95.2%.

### Development and application of the CS-nomogram

**Figure 3:**
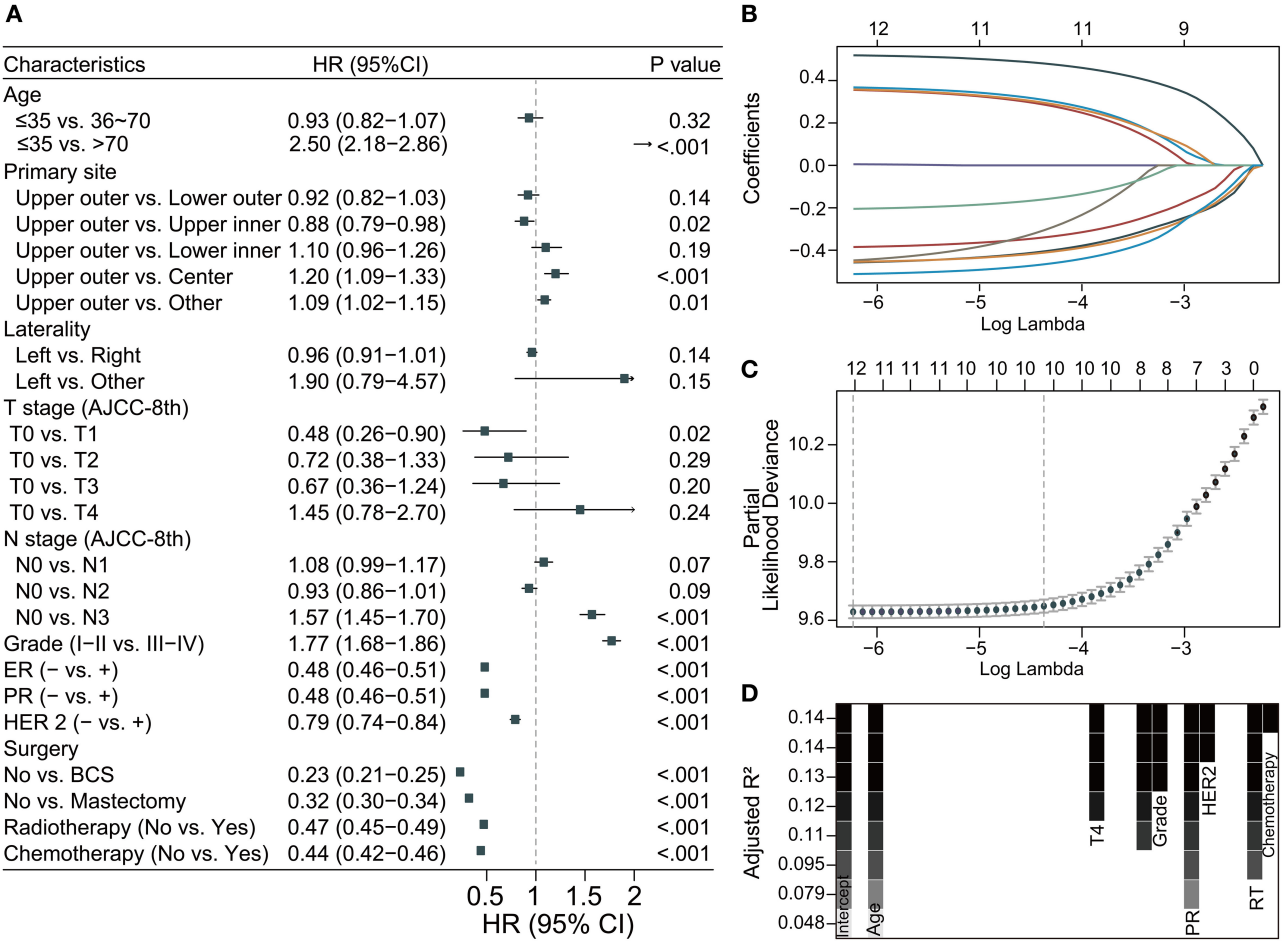
Predictor screening. **(A)** Univariate Cox regression; **(B,C)** the least absolute shrinkage and selection operator (LASSO) regression and 5-fold cross-validation; **(D)** best subset regression (BSR). ER, estrogen receptor; PR, progesterone receptor; HER 2, human epidermal growth factor receptor 2; BCS, breast conservation surgery; HR, hazard ratio; 95% CI, 95% confidence interval; RT, radiation therapy.

Based on the training data, the three methods used to find predictors for identifying OS in LABC were shown in Figure 3. The forest plot showed that a total of 11 (11/12) factors were selected based on univariate Cox regression (all *p*-values were < 0.05) (Figure 3A). In the LASSO regression, the log lambda value corresponding to the minimum mean squared error plus one standard error was −3.81, and a total of 10 (10/12) factors were thus selected (Figures 3B,C). An 8-variable (8/12) combination was found based on the adjusted R-squared maximum of the BSR (Figure 3D). The respective combinations of variables selected for the three methods were summarized in Supplementary Table 1. Finally, after reconfirmation by multivariate Cox stepwise backward regression, the subset of 10 variables screened by the LASSO regression was the best model (AIC = 106353.5, R-square = 0.183), and the results of the univariate Cox analysis with stepwise backward regression also came to the same conclusion. The 10 variables included age, T status, N status, grade, estrogen receptor (ER) status, progesterone receptor (PR) status, human epidermal growth factor receptor 2 (HER 2), surgery, radiotherapy, and chemotherapy, and the results of multivariate Cox regression were shown in Supplementary Table 2.

**Figure 4:**
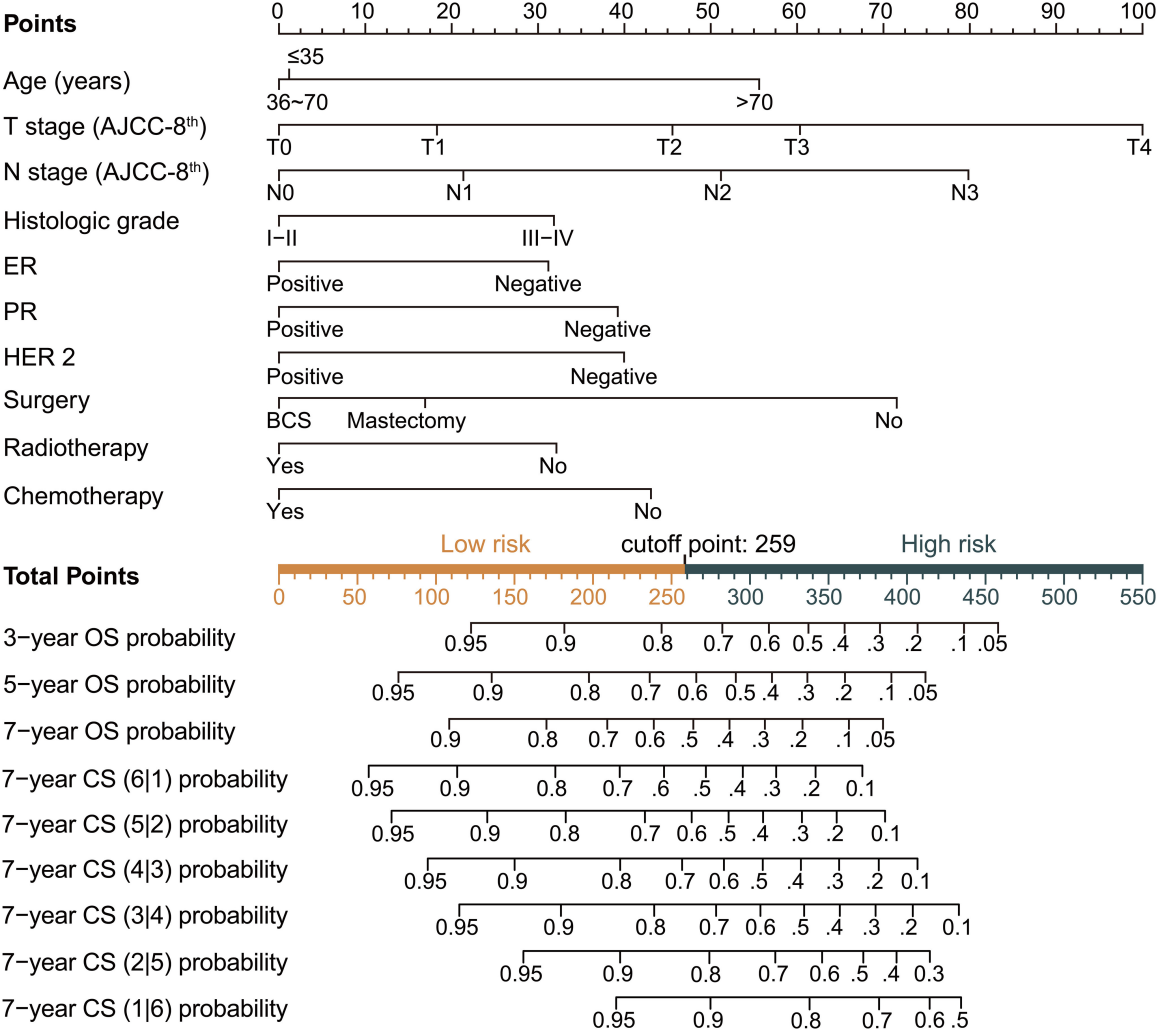
Dynamic condition survival nomogram for predicting overall survival (OS) and conditional survival (CS) for patients with locally advanced breast cancer (LABC). (1) When using the nomogram, 10 predictors were quantified as “point” based on patient-specific factors and then the sum of the “point” corresponded to the “total point” below, which corresponded to the 3, 5, 7 year OS and 7 year CS; (2) The web version of the nomogram was available at: https://impcofmxd.shinyapps.io/LABC/; (3) The optimal cut-off total point calculated from the maximum standardized log-rank statistic was 259, which divided the patients into high-risk group and low-risk group.

**Figure 5:**
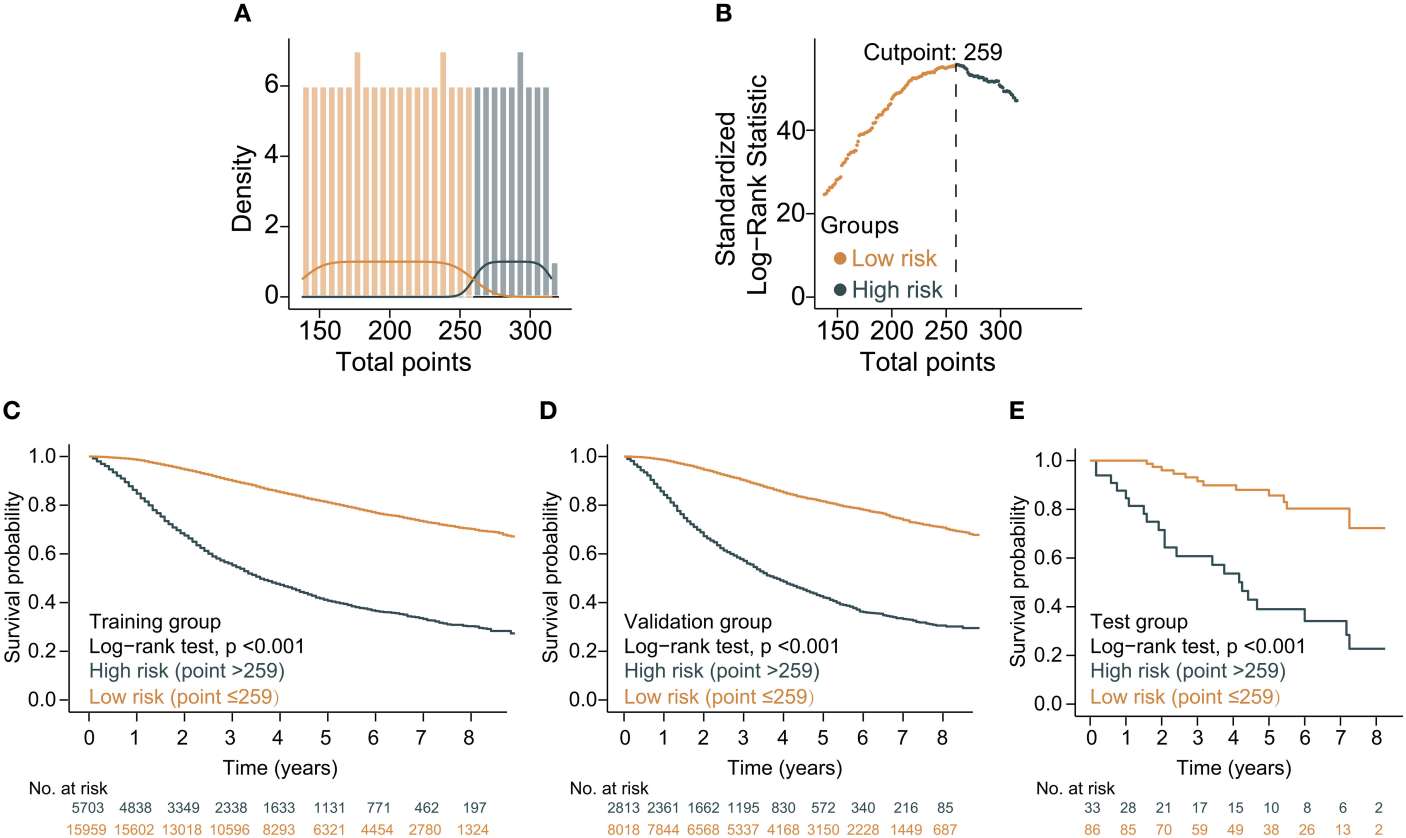
Construction and validation of risk stratification based on the total points of the condition survival nomogram. **(A)** Distribution of total risk points; **(B)** The standardized log-rank statistics; **(C–E)** Kaplan–Meier for estimating risk stratification in training, validation, and testing groups, respectively.

Based on the above variables and the CS formula, we constructed a dynamic CS-nomogram that could assess the 3, 5, and 7 year OS and 7 year CS for LABC patients (Figure 4), and its web version could be found at https://impcofmxd.shinyapps.io/LABC/. When using this model, the clinicopathological characteristics were first entered, and they were converted to points in the nomogram. Then, the sum of all the points was calculated to obtain the total risk points, under which corresponded the patient survival and CS. Based on this nomogram, risk points were calculated for LABC patients in the training group. The maximum standardized log-rank statistic for survival at this risk point was 55.81, which estimated the optimum cut-off point as 259 (Figures 5A,B). The Kaplan–Meier curves for training group, validation and testing groups showed that patients in the low-risk group (total points ≤259) had a better survival advantage than those in the high-risk group (total points >259) (log-rank test, *p* < 0.001) (Figures 5C–E).

### Evaluation and validation

**Figure 6:**
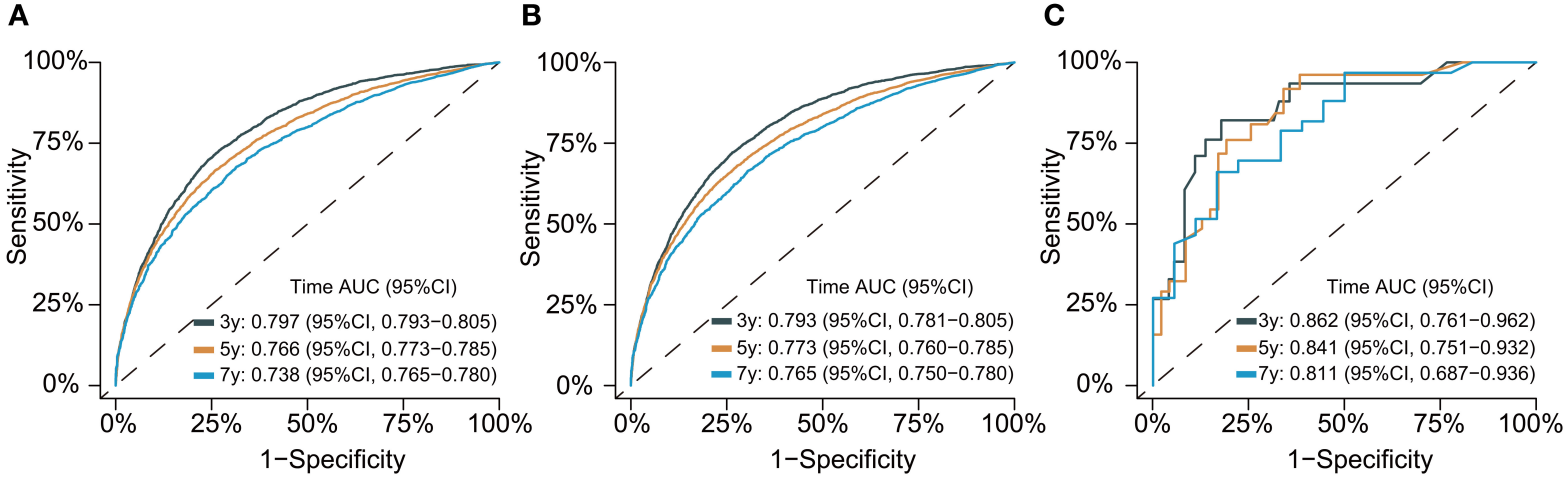
Time-dependent receiver operating characteristic (ROC) curves for assessing the discrimination of the condition survival nomogram in training **(A)**, validation **(B)**, and testing groups **(C)**, respectively. AUC, area under the curve.

**Figure 7:**
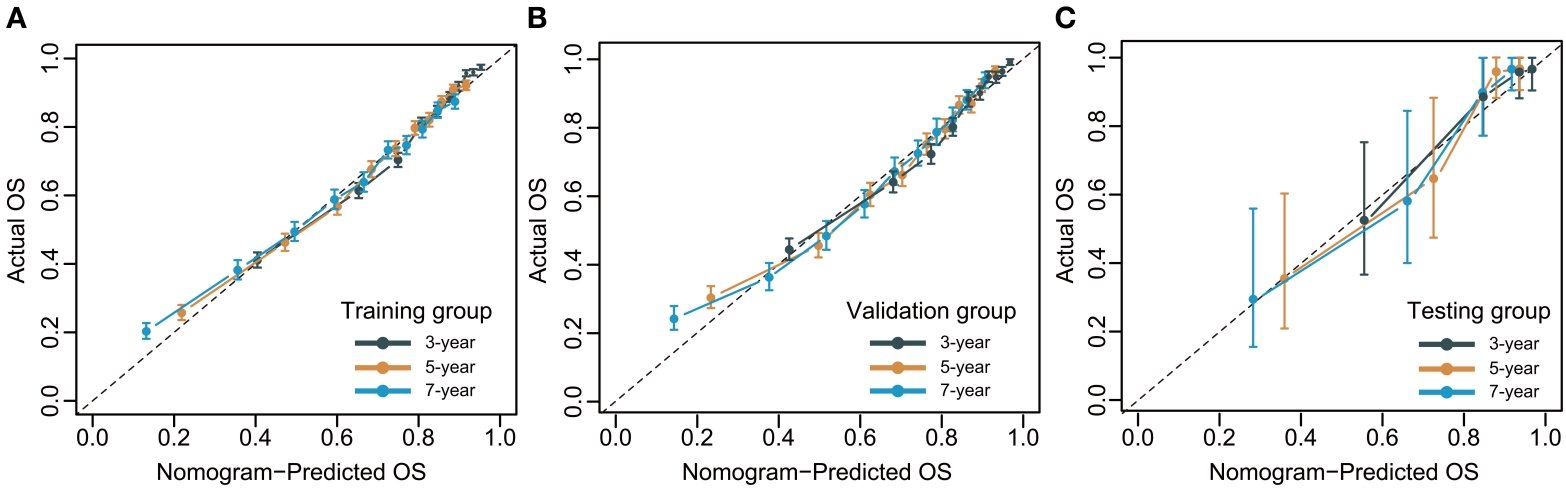
Calibration plots for 3-, 5- and 7-year overall survival (OS) of condition survival nomogram in training **(A)**, validation **(B)**, and testing groups **(C)**.

**Figure 8:**
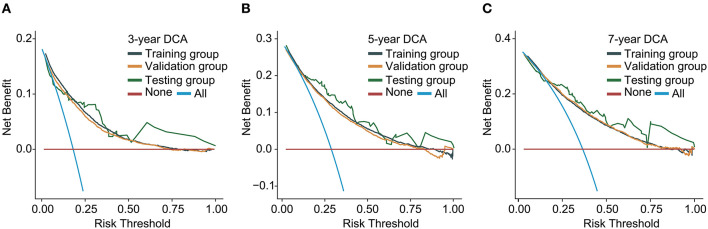
Decision curve analysis (DCA) curves for assessing clinical usefulness of the condition survival nomogram in training **(A)**, validation **(B)**, and testing groups **(C)**.

The C-index for the dynamic CS-nomogram was 0.761 (95% CI: 0.755–0.767), 0.768 (95% CI: 0.757–0.773), and 0.810 (95% CI: 0.736–0.884) in the training, validation and testing groups. Time-dependent ROCs at 3, 5, and 7 year indicated that this model was sufficiently differentiable, with areas under the curves (AUCs) of 0.797 (95% CI: 0.793–0.805), 0.766 (95% CI: 0.773–0.785), and 0.738 (95% CI: 0.750–0.780) in the training group, 0.793 (95% CI: 0.781–0.805), 0.773 (95% CI: 0.760–0.785), and 0.765 (95% CI: 0.728–0.750) in the validation group and 0.862 (95% CI: 0.761–0.962), 0.841 (95% CI: 0.751–0.932), and 0.811 (95% CI: 0.687–0.936) in the testing group (Figure 6). The calibration plots from the training, validation and testing groups showed that this model was well-calibrated to reality, with the curves close to the 45-degree line (Figure 7). Meanwhile, DCA curves showed a good net benefit if LABC patients used the CS-nomogram as a guide for medical intervention (Figure 8). The benefit range of risk threshold probabilities of the CS-nomogram estimated in the training, validation and testing groups were 0.03–0.74, 0.08–0.71, and 0.11–1.00 at 3 years, 0.04–0.84, 0.12–0.77, and 0.00–1.00 at 5 years, and 0.04–0.84, 0.00–0.78, and 0.00–1.00 at 7 years, respectively.

## Discussion

This first study of CS in LABC found that the long-term survival of LABC improved dynamically over time. Meanwhile, after 2 years of follow-up, for each additional year the patients lived, the survival for the next year was significantly improved. Furthermore, we combined CS analysis and prognostic correlations to develop the first CS-nomogram for predicting OS in LABC and its web version. Based on this model, we could accurately calculate survival after surviving for several years. In addition, the risk stratification constructed from the risk points could further guide follow-up and treatment. The excellent performance of the CS-nomogram was well-evaluated and validated in the training, validation, and testing groups.

Our study suggested that the survival in LABC patients was not consistently unsatisfactory, with a 5 year OS of ~40–75% as described in previous studies (2, 3). CS was a novel survival assessment method that allowed real-time estimation of changes in survival based on survived time (9). More recently, this method has been widely used in clinical trials [e.g., colon cancer (17), gastrointestinal stromal tumors (26), and melanoma (10)] to analyse changes in the distribution of survival with disease progresses under the CS. We found that despite the relatively advanced stage of LABC, long-term survival improved significantly for each additional year patients lived. It has been noted in previous studies of the National Surgical Adjuvant Breast and Bowel Project (NSABP) pilot studies that the harmfulness of certain adverse factors for patients decreases over time (17, 27). Another reason for the changing survival dynamics may be the effort of natural selection, in which high-risk patients die within the first few years and the remaining low-risk patients may have a better prognosis (17). Furthermore, these beneficial changes may bring more hope to survivors, and they will feel more reassured as their survival estimates continue to improve. And a positive psychological state may lead to greater adherence to follow-up and treatment, ultimately improving survival.

For LABC, 1 year CS decreased significantly in the 2nd year after diagnosis [CS(1|1) = 92.2%], but this level gradually improved in subsequent years. The decline in survival at year 2 after diagnosis not only reflected the non-linear OS of LABC, but also implied that additional attention or treatment may be required for high-risk patients despite the end of primary tumor treatment after year 1. Although the exact reason for the decline in CS(1|1) was unknown, accurately identifying of such high-risk patients would help guide follow-up and treatment. In addition, when patients' survival rate was 95%, it was reasonable to assume that there was little additional mortality in the patients (survival was indistinguishable from the general population of the same age), a conclusion that has been confirmed in the studies of colorectal, cervical, gastric, and endometrial cancers (4). This improvement in LABC occurred in the 7th year after diagnosis [CS(1|7) = 95.2%], which meant that assuming patients lived to 7 years, their risk of death in the 8th year was no different from that of general people. Therefore, for LABC patients, a follow-up period of at least 7 years may be required. Compared to traditional survival analysis, CS contained more real-time and accurate clinical prognostic information, which became more valuable for patient expectations, physician follow-up, and clinical trials of new therapies expected to increase survival.

The real-time prognosis of patients was not only dynamically related to time, but also varied according to individual clinicopathological characteristics (28, 29), and the dynamic CS-nomogram was developed based on this. To avoid overfitting or underfitting the model, we tried to determine the best model using Cox regression, BSR and the LASSO regression. After rigorous screening and comparison, this novel model used the current widely accepted LABC predictors. Considering individualized prediction, we used T and N status separately in the nomogram because patients with different T and N statuses may have different prognoses even with the same TNM stage. The advantage of our model over published nomograms was that it took the survived time into account, and we developed a web version for ease of calculation and use. Furthermore, risk stratification based on risk points was a major application of this model. Patients in the high-risk group of LABC may receive more frequent follow-up or participate in clinical trials of novel agents that may increase survival. To our knowledge, no nomogram for LABC has been established, so we cannot use the same type of nomogram for comparison. However, the nomogram achieved good accuracy and stability across training, validation and testing groups, and its C-index (0.761) was also higher than the median value (0.74) reported in published prediction models (28). What's more, the external validation of the nomogram using data from the Oriental Medicine Research Centre added to its credibility, and the test results for this test data were excellent. In conclusion, our model had both dynamic and personalized predictive capabilities, which greatly improved the prediction accuracy. Based on this model, reducing the psychological burden of patients, thereby improving treatment and follow-up compliance, researchers will gain more valuable information to design clinical trials.

This study has some limitations. First, as a retrospective study, some potential selection bias is unavoidable. Second, despite the rich sample size of the SEER database, the lack of information on lymphatic vascular invasion, margin status, Ki-67 and specific treatment plans limited some of our analyses. Although the nomogram had a C-index of 0.81 in the test group, the differences in clinicopathological characteristics and treatments between the Eastern and Western patients shown in Table 1 suggest that this may have influenced the results. Third, the number of patients available for CS analysis has decreased over time, which may affect prediction accuracy in the final years. However, due to the large initial follow-up data, the time-dependent AUC showed that the discrimination of the model did not decrease much. Fourth, although the study concluded that traditional prognostic and predictive factors may remain valid for LABC (1), future use of new therapies may lead to improved survival in LABC and predicted survival may be underestimated.

## Conclusions

CS analysis revealed a dynamic and non-linear improvement in real-time survival of LABC patients over time. Based on CS and several significant predictors, we developed the first CS-nomogram with a web version to predict the dynamic survival and established risk stratification. This model provided accurate and real-time prognostic information that would develop more personalized and cost-effective follow-up strategies and provide necessary treatment recommendations for patients, as well as alleviate patients' pressure by providing real and progressively improving survival outcomes.

## Data availability statement

The raw data supporting the conclusions of this article will be made available by the authors, without undue reservation.

## Ethics statement

Ethical review and approval was not required for the study on human participants in accordance with the local legislation and institutional requirements. Written informed consent for participation was not required for this study in accordance with the national legislation and the institutional requirements.

## Author contributions

XM and YG designed the study. XM, ZJ, and FH performed the study and analyzed the data. XM wrote the manuscript. XM and FH provided the expert consultations and clinical suggestions. XC and ZJ conceived of the study, participated in its design, and coordination. ZJ, XC, and YG helped to draft the manuscript. All authors contributed to the article and approved the submitted version.

## Conflict of interest

The authors declare that the research was conducted in the absence of any commercial or financial relationships that could be construed as a potential conflict of interest.

## Publisher's note

All claims expressed in this article are solely those of the authors and do not necessarily represent those of their affiliated organizations, or those of the publisher, the editors and the reviewers. Any product that may be evaluated in this article, or claim that may be made by its manufacturer, is not guaranteed or endorsed by the publisher.

## References

[1] Aebi S Karlsson P Wapnir IL. Locally advanced breast cancer. Breast. (2021) 62(Suppl. 1):S58–62. 10.1016/S0960-9776(21)00086-2 34930650 PMC9097810

[2] Dhanushkodi M Sridevi V Shanta V Rama R Swaminathan R Selvaluxmy G. Locally advanced breast cancer (LaBc): real-world outcome of patients from Cancer Institute, Chennai. JCO Glob Oncol. (2021) 7:767–81. 10.1200/GO.21.00001 34043414 PMC8457812

[3] Simos D Clemons M Ginsburg OM Jacobs C. Definition and consequences of locally advanced breast cancer. Curr Opin Support Palliat Care. (2014) 8:33–8. 10.1097/SPC.0000000000000020 24270749

[4] Janssen-Heijnen ML Gondos A Bray F Hakulinen T Brewster DH Brenner H. Clinical relevance of conditional survival of cancer patients in europe: age-specific analyses of 13 cancers. J Clin Oncol. (2010) 28:2520–8. 10.1200/JCO.2009.25.9697 20406936

[5] Zhong Q Chen QY Li P Xie JW Wang JB Lin JX. Prediction of conditional probability of survival after surgery for gastric cancer: a study based on eastern and western large data sets. Surgery. (2018) 163:1307–16. 10.1016/j.surg.2018.02.011 29685636

[6] Shah MM Meyer BI Rhee K NeMoyer RE Lin Y Tzeng CD. Conditional survival analysis of hepatocellular carcinoma. J Surg Oncol. (2020). 10.1002/jso.26049 32524634 PMC8565605

[7] Hagens ERC Feenstra ML Eshuis WJ Hulshof M van Laarhoven HWM van Berge Henegouwen MI. Conditional survival after neoadjuvant chemoradiotherapy and surgery for oesophageal cancer. Br J Surg. (2020) 107:1053–61. 10.1002/bjs.11476 32017047 PMC7317937

[8] Miura JT Lindner H Karakousis GC Sharon CE Gimotty PA. Conditional survival estimates for merkel cell carcinoma reveal the dynamic nature of prognostication. J Surg Oncol. (2022) 126:348–55. 10.1002/jso.26861 35315930

[9] Jung SH Lee HY Chow SC. Statistical methods for conditional survival analysis. J Biopharm Stat. (2018) 28:927–38. 10.1080/10543406.2017.1405012 29185865 PMC6195126

[10] Zabor EC Gonen M Chapman PB Panageas KS. Dynamic prognostication using conditional survival estimates. Cancer. (2013) 119:3589–92. 10.1002/cncr.28273 23913639

[11] Hieke S Kleber M König C Engelhardt M Schumacher M. Conditional survival: a useful concept to provide information on how prognosis evolves over time. Clin Cancer Res. (2015) 21:1530–6. 10.1158/1078-0432.CCR-14-2154 25833308

[12] Wang J Huang X Sun S Wang K Qu Y Chen X. Stage-dependent conditional survival and failure hazard of non-metastatic nasopharyngeal carcinoma after intensity-modulated radiation therapy: clinical implications for treatment strategies and surveillance. Cancer Med. (2021) 10:3613–21. 10.1002/cam4.3917 33960136 PMC8178506

[13] Ke B Cai X Peng X. Survival prediction and conditional survival of primary central nervous system lymphoma: a population-based study. J Clin Neurosci. (2021) 93:188–94. 10.1016/j.jocn.2021.09.026 34656246

[14] Yoo JE Han K Shin DW Park SH Cho IY Yoon DW. Conditional relative survival and competing mortality in patients who underwent surgery for lung cancer: a nationwide cohort study. Int J Cancer. (2021) 148:626–36. 10.1002/ijc.33239 32738818

[15] Shin DW Cho JH Yoo JE Cho J Yoon DW Lee G. Conditional survival of surgically treated patients with lung cancer: a comprehensive analyses of overall, recurrence-free, and relative survival. Cancer Res Treat. (2021) 53:1057–71. 10.4143/crt.2020.1308 33705624 PMC8524014

[16] Shin DW Kim HK Cho J Lee G Cho J Yoo JE. Conditional survival of patients who underwent curative resection for esophageal squamous cell carcinoma. Ann Surg. (2020) 276:e86–92. 10.1097/SLA.0000000000004473 33086317 PMC9259045

[17] Zamboni BA Yothers G Choi M Fuller CD Dignam JJ Raich PC. Conditional survival and the choice of conditioning set for patients with colon cancer: an analysis of Nsabp trials C-03 through C-07. J Clin Oncol. (2010) 28:2544–8. 10.1200/JCO.2009.23.0573 20406942 PMC2881729

[18] Zheng Z Wang X Liu Z Lu X Huang Y Chi P. Individualized conditional survival nomograms for patients with locally advanced rectal cancer treated with neoadjuvant chemoradiotherapy and radical surgery. Eur J Surg Oncol. (2021) 47:3175–81. 10.1016/j.ejso.2021.06.010 34120806

[19] Latenstein AEJ van Roessel S van der Geest LGM Bonsing BA Dejong CHC Groot Koerkamp B. Conditional survival after resection for pancreatic cancer: a population-based study and prediction model. Ann Surg Oncol. (2020) 27:2516–24. 10.1245/s10434-020-08235-w 32052299 PMC7311496

[20] Singletary SE Allred C Ashley P Bassett LW Berry D Bland KI. Revision of the American Joint Committee on cancer staging system for breast cancer. J Clin Oncol. (2002) 20:3628–36. 10.1200/JCO.2002.02.026 12202663

[21] Schrijvers D Aapro M Zakotnik B Audisio R. ESMO Handbook of Cancer in the Senior Patient, 1st Edn. CRC Press (2010). p. 1–7. 10.3109/9781841847481

[22] Xu Z Wang L Dai S Chen M Li F Sun J. Epidemiologic trends of and factors associated with overall survival for patients with gastroenteropancreatic neuroendocrine tumors in the United States. JAMA Netw Open. (2021) 4:e2124750. 10.1001/jamanetworkopen.2021.24750 34554237 PMC8461504

[23] Lyu MH Ma YZ Tian PQ Guo HH Wang C Liu ZZ. Development and validation of a nomogram for predicting survival of breast cancer patients with ipsilateral supraclavicular lymph node metastasis. Chin Med J. (2021) 134:2692–9. 10.1097/CM9.0000000000001755 34743149 PMC8631408

[24] Deng X Hou H Wang X Li Q Li X Yang Z. Development and validation of a nomogram to better predict hypertension based on a 10-year retrospective cohort study in China. eLife. (2021) 10:e66419. 10.7554/eLife.66419 34047697 PMC8163499

[25] Yuan K Chen J Xu P Zhang X Gong X Wu M. A nomogram for predicting stroke recurrence among young adults. Stroke. (2020) 51:1865–7. 10.1161/STROKEAHA.120.029740 32390546

[26] Bischof DA Kim Y Dodson R Jimenez MC Behman R Cocieru A. Conditional disease-free survival after surgical resection of gastrointestinal stromal tumors: a multi-institutional analysis of 502 patients. JAMA Surg. (2015) 150:299–306. 10.1001/jamasurg.2014.2881 25671681 PMC4703090

[27] Bryant J Fisher B Gündüz N Costantino JP Emir B. S-Phase fraction combined with other patient and tumor characteristics for the prognosis of node-negative, estrogen-receptor-positive breast cancer. Breast Cancer Res Treat. (1998) 51:239–53. 10.1023/A:1006184428857 10068082

[28] Balachandran VP Gonen M Smith JJ DeMatteo RP. Nomograms in oncology: more than meets the eye. Lancet Oncol. (2015) 16:e173–80. 10.1016/S1470-2045(14)71116-7 25846097 PMC4465353

[29] Iasonos A Schrag D Raj GV Panageas KS. How to build and interpret a nomogram for cancer prognosis. J Clin Oncol. (2008) 26:1364–70. 10.1200/JCO.2007.12.9791 18323559

